# Research progress on the mechanisms of interleukin and chemokine families in driving calcium oxalate nephrolithiasis formation

**DOI:** 10.3389/fimmu.2025.1651003

**Published:** 2025-09-12

**Authors:** Fujie Liang, Fuyou Guo, Runjun Liu, Xiang Wang

**Affiliations:** Department of Urology, The First Affiliated Hospital of Guangxi Medical University, Nanning, China

**Keywords:** kidney stone, interleukin, chemokines, inflammation, renal fibrosis

## Abstract

Calcium Oxalate Nephrolithiasis is a globally prevalent urological disorder, with its pathogenesis involving multiple mechanisms such as inflammatory responses, oxidative stress, crystal-cell interactions, macrophage polarization, and fibrosis. In recent years, the multidimensional regulatory roles of interleukins (ILs) and chemokines in stone formation have garnered increasing attention. Pro-inflammatory interleukins, such as IL-1β, may promote crystal deposition, oxidative stress, and renal tubular epithelial cell injury by activating signaling pathways including NLRP3 inflammasome, NF-κB, and MAPK. In contrast, anti-inflammatory interleukins, by stimulating M2 macrophage polarization and suppressing crystal adhesion and oxidative damage, exhibit nephroprotective effects. Notably, IL-6 demonstrates unique bidirectional regulatory properties. Chemokines play critical roles in recruiting immune cells, amplifying inflammatory responses, modulating crystal-cell interactions, and sustaining the fibrosis-stone vicious cycle. The CXCL12/CXCR4 axis has emerged as a potential hub in regulating crystal autophagy and fibrotic progression. Additionally, miR-124-3p overexpression inhibits pro-inflammatory factor expression and promotes M2 macrophage polarization, while the IL-6/MCP-1 axis may reverse this suppression via a negative feedback network. This review integrates the multidimensional regulatory mechanisms of interleukins and chemokines in Calcium Oxalate Nephrolithiasis and proposes three novel hypotheses: the dynamic regulatory model of IL-6, the MCP-1-mediated fibrosis-stone vicious cycle, and the IL-6/MCP-1/miR-124-3p negative feedback loop.

## 1 Introduction

Kidney stones are a global health burden, with 80%–90% of cases being calcium oxalate (CaOx) stones (1). The incidence and recurrence rates of nephrolithiasis have risen due to modern lifestyle changes and global warming. In China, approximately 1 in 17 adults suffers from kidney stones, with males aged 31–60 exhibiting significantly higher prevalence than females (2–4). The formation of kidney stones is a multi-step pathophysiological process, triggered by a pathological imbalance in the urinary environment, influenced by cell damage, and regulated by genetic and metabolic factors (5). Under supersaturated conditions caused by excessive intake of solutes, dehydration, or metabolic abnormalities, compounds such as calcium, oxalate, or uric acid form microcrystalline nuclei that precipitate in the renal tubules. These crystals typically nucleate on Randall’s plaques, which are deposits of calcium phosphate located on the basement membrane of the renal papilla. Additionally, crystal-induced cell damage activates pathogenic pathways involving oxidative stress and mitochondrial dysfunction, leading to the release of extracellular vesicles and adhesion molecules (5, 6). Overall, these mechanisms promote crystal aggregation and subsequent stone growth.

There are mainly five types of kidney stones: calcium oxalate, calcium phosphate, uric acid, magnesium ammonium phosphate and cystine. Among them, calcium oxalate kidney stones are the most common. The formation of Calcium Oxalate Nephrolithiasis involves diverse physiological and chemical processes, including urine composition alterations, oxidative stress, inflammatory responses, macrophage infiltration, autophagy, gut microbiota dysbiosis, and renal fibrosis (7–11). The pathophysiological process of calcium oxalate kidney stones stems from the nucleation and deposition of calcium oxalate crystals in the renal tubules, mainly caused by the supersaturation of calcium oxalate in urine (12). This supersaturated state promotes the precipitation, growth and aggregation of crystals, eventually forming the initial center point of stone formation.These crystals attach to the epithelial cells of renal tubules, triggering oxidative stress and activating multiple signaling pathways such as NLRP3 inflammasome, TLR4/NF-κB, and p38 MAPK, resulting in the massive release of inflammatory cytokines and amplification of local inflammatory responses (12, 13). Meanwhile, the immunomodulatory mechanism induces macrophages to polarize towards the M1 phenotype, jointly promoting the occurrence and development of calcium oxalate kidney stones (14).

Emerging evidence suggests that inflammation is not merely a consequence but an active driver of CaOx stone formation (15, 16). Interleukins and chemokines are markedly upregulated in renal tubular epithelial cells and stone microenvironments, highlighting their critical roles in pathogenesis. While previous reviews focused on their roles in inflammation and oxidative stress (**Table 1**), this article systematically integrates their regulatory networks and elucidates their interplay with fibrosis, offering new insights into the dynamic interactions between inflammatory drivers and renal structural remodeling.

**Table 1:** Regulatory mechanisms of interleukin and chemokine families in Calcium Oxalate Nephrolithiasis: recent advances (9, 16, 87–91).

Title	Year of publication	Key conclusions
《Roles Played by Biomarkers of Kidney Injury in Patients with Upper Urinary Tract Obstruction》	2020	The up-regulation of inflammatory factors such as MCP-1, TNF-α, and TGF-β1, as well as the increase in ROS production, jointly promoted the development of calcium oxalate crystal kidney injury and fibrosis.
《Randall's plaque and calcium oxalate stone formation: role for immunity and inflammation》	2021	The release of inflammatory factors such as IL-1β, IL-6, and TNF-α will exacerbate local inflammation and promote the formation and growth of crystals, while inflammation interacts with oxidative stress to form a vicious circle.
《Inflammation and kidney stones: cause and effect?》	2022	Activation of immune cells, assembly of NLRP3 inflammasomes, and release of pro-inflammatory cytokines such as IL-1β, IL-18, and IL-6 lead to tissue damage and further stone formation, and oxidative stress, as a link in the inflammatory response, is also involved in this process.
《Proposal for pathogenesis-based treatment options to reduce calcium oxalate stone recurrence》	2023	IL-1β, IL-18; CCL2, CCL5, CCL7 and other cytokines trigger and exacerbate inflammatory responses, creating conditions for the formation of stones.
《New insight into oxidative stress and inflammatory responses to kidney stones: Potential therapeutic strategies with natural active ingredients》	2024	Natural active ingredients prevent and treat kidney stones by modulating inflammatory and oxidative stress related pathways, the mechanism of which involves inhibition of the release of pro-inflammatory factors such as IL-1β, IL-6, TNF-α etc.
《Recent developments in the study of cellular inflammation in the papillae of stone formers》	2025	Inflammation plays an important role in kidney stone formation and kidney stone disease, but different types of kidney stones and different stone formation mechanisms may involve different inflammatory pathways.
《Molecular insights into cell signaling pathways in kidney stone formation 》	2025	Calcium oxalate crystals activate the NLRP3 inflammasome, leading to the release of pro-inflammatory factors such as IL-1β and increased production of reactive oxygen species (ROS), triggering oxidative stress and promoting the formation of kidney stones.

Previous studies have shown that the inflammatory and oxidative stress effects guided by the interleukin family and chemokine family can promote the formation of calcium oxalate stones in the kidneys to a certain extent.

## 2 Dual regulatory roles of interleukins in calcium oxalate nephrolithiasis

Interleukins(ILs), secreted by immune and non-immune cells, are pleiotropic cytokines central to innate immunity, inflammation, and tissue homeostasis. Based on functional characteristics, ILs are classified into pro-inflammatory (e.g., IL-1β) and anti-inflammatory (e.g., IL-10) subgroups. Recent studies reveal their pivotal roles in CaOx stone formation through inflammation, oxidative stress, and crystal-cell interactions.

### 2.1 IL-1β/IL-6-mediated inflammation and oxidative stress promote CaOx stone formation

#### 2.1.1 Inflammatory roles and molecular mechanisms of IL-1β and IL-6

**Figure 1:**
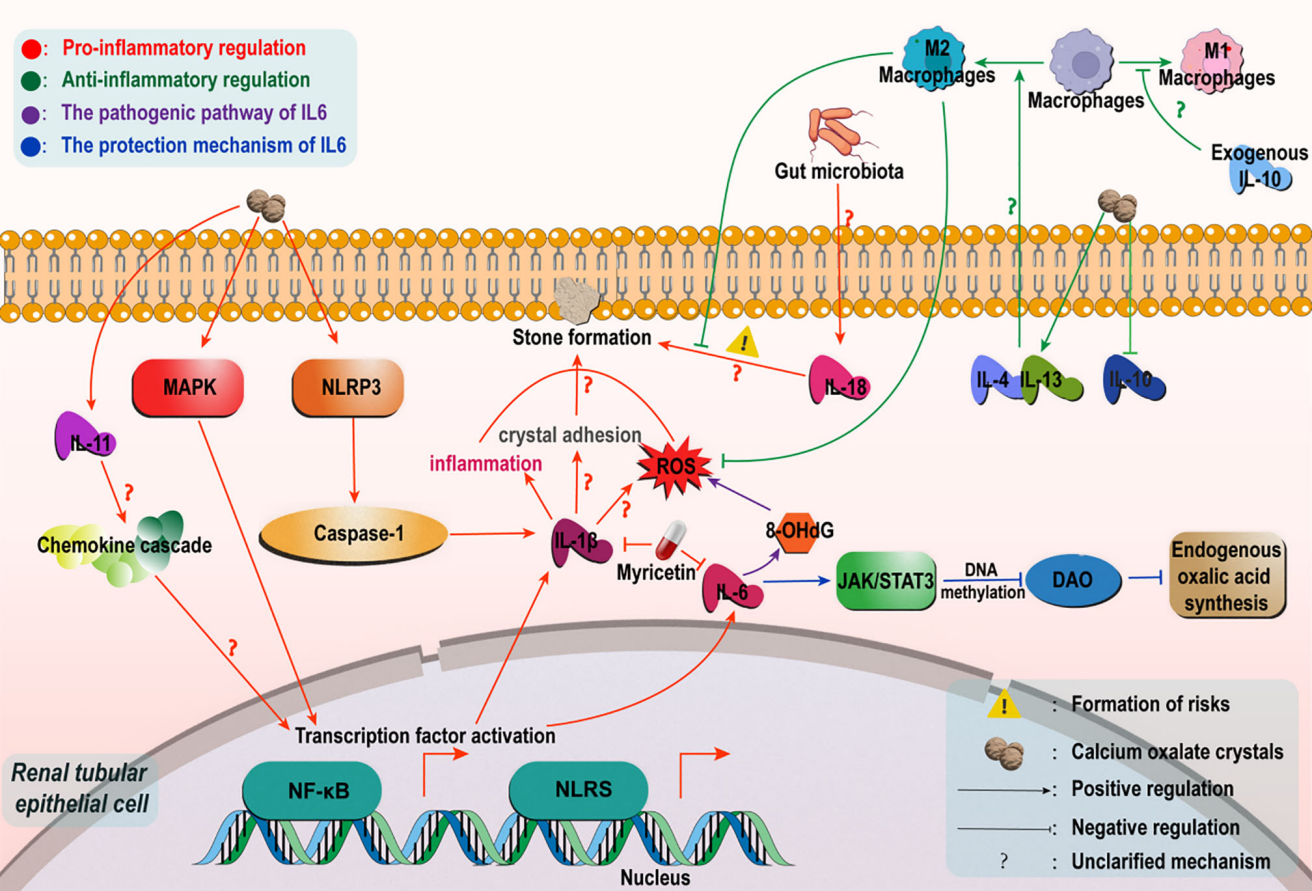
Regulatory mechanisms of the interleukin family in Calcium Oxalate Nephrolithiasis. In a high oxalic acid environment, calcium oxalate crystals mediate caspase-1-dependent IL-1βrelease by activating the NLRP3 inflammatome, trigger tubular inflammatory injury and ROS generation, and synergize with the NF-κB/MAPK pathway to enhance crystal adhesion and deposition. IL-6 has a biphasic effect: In the early stage, it inhibits DAO through the IL-6/JAK/STAT3 pathway to reduce oxalic acid synthesis; In the later stage, ROS/NF-κB is activated to intensify oxidative stress and adhesion. Among non-classical factors, IL-11 is involved in stone formation through chemokine/TLR/NLR signaling. Dysbiosis of the intestinal flora upregulates IL-18 via the gut-renal axis to increase susceptibility. In terms of anti-inflammation, IL-4/IL-13 promotes M2-polarized phagocytosis crystals in macrophages, and IL-10 can improve immune dysfunction induced by high oxalic acid.

At the regulatory level of pro-inflammatory cytokines, interleukin-1β (IL-1β) and interleukin-6 (IL-6), as core mediators of inflammatory responses, play pivotal regulatory roles in Calcium Oxalate Nephrolithiasis formation (**Figure 1**). Substantial evidence indicates significant upregulation of IL-1β and IL-6 expression levels during nephrolithiasis development, particularly in calcium oxalate stone formation (17). The high-oxalate microenvironment and calcium oxalate crystals activate the NLRP3 inflammasome, leading to caspase-1-dependent maturation and release of IL-1β, which subsequently drives inflammatory injury in renal tubular epithelial cells and promotes reactive oxygen species (ROS) generation (18, 19). Furthermore, emerging studies suggest that the elevated expression of IL-1β and IL-6 in Calcium Oxalate Nephrolithiasis may also depend on NF-κB and MAPK signaling pathway regulation (20, 21). Notably, the upregulated IL-1β expression demonstrates a significant positive correlation with enhanced calcium oxalate crystal adhesion capacity, suggesting its potential role in mediating intrarenal crystal deposition through strengthening crystal-cell interfacial interactions (17), thereby facilitating calcium oxalate stone pathogenesis.

Recent advances in therapeutic strategies have demonstrated that targeted inhibition of pro-inflammatory cytokines IL-1β and IL-6 expression can effectively intervene in calcium oxalate stone formation (**Figure 1**). For instance, myricetin exhibits anti-nephrolithic effects by suppressing IL-1β and IL-6 release, consequently alleviating renal stone formation and nephrotoxicity (22). Similarly, downregulation of IL-6 expression significantly inhibits oxalate-induced oxidative stress and inflammatory damage, thereby reducing lithogenic propensity (23). These collective findings substantiate that abnormal activation of the IL-1β/IL-6 signaling axis constitutes a critical mechanism underlying inflammatory injury and oxidative stress initiation during calcium oxalate calculogenesis. Concurrently, this pathway enhances crystal-cell adhesion interactions, creating a permissive microenvironment for calcium oxalate renal stone development.

#### 2.1.2 Dual regulatory dynamics of IL-6: protective suppression versus pro-inflammatory aggravation

While both IL-6 and IL-1β are canonical pro-inflammatory cytokines, their mechanistic roles in Calcium Oxalate Nephrolithiasis exhibit distinct divergence (**Table 2**). Notably, IL-1β demonstrates consistent pro-lithogenic effects, whereas IL-6 displays paradoxical regulatory duality in calcium oxalate stone pathogenesis. Recent convergent validation across multi-species models (Drosophila, murine, and HK-2 cells) revealed that IL-6/JAK/STAT3 pathway activation epigenetically suppresses D-amino acid oxidase (DAO) transcription via DNA methylation modifications, thereby attenuating endogenous oxalate biosynthesis and mitigating hyperoxaluria risks. This underscores the evolutionary conservation and functional universality of the IL-6/JAK/STAT3 signaling axis (24). Paradoxically, in ethylene glycol-induced rat models of Calcium Oxalate Nephrolithiasis, elevated IL-6 levels exhibit significant positive correlation with oxidative stress marker 8-OHdG, implicating its role in promoting crystal deposition through ROS-mediated cascades (23) (**Figure 1**). To reconcile this functional dichotomy, we postulate a temporal regulatory hypothesis: During acute oxalate exposure, IL-6 may exert protective effects by suppressing DAO-driven oxalate synthesis via IL-6/JAK/STAT3 activation; conversely, in chronic lithogenic phases, sustained inflammatory microenvironments potentially drive IL-6-mediated ROS/NF-κB pathway activation, exacerbating oxidative stress and crystal adhesion. And the duration of calcium oxalate crystal exposure may be the underlying factor that triggers the transition between these two mechanisms. This paradigm highlights the necessity for stage-specific therapeutic targeting of IL-6 signaling. Future investigations should prioritize developing phase-defined IL-6 knockout models (e.g., conditional IL-6 depletion in acute oxalate challenge versus chronic stone-forming stages) to elucidate the microenvironment-dependent functional equilibrium governing IL-6’s dual roles.

**Table 2:** Bidirectional effects of IL-6 in Calcium Oxalate Nephrolithiasis formation (23, 24).

Contents	Anti-calculative effect of IL-6	The lithophilic effect of IL-6
Model	Mice	Rats
Inducing medications	Glyoxylate (GA)	Ethylene glycol(EG)and ammonium chloride(AC)
Mode of intervention	Mice were administered 75 mg/kg body weight of glyoxonic acid by intraperitoneal injection for 6 consecutive days.	EG is provided as drinking water for 3 weeks. AC is given by gavage for the first three days of the first and third weeks at 2 mL/animal per day.
Signaling pathways	IL-6/JAK/STAT3 activates → inhibits DAO expression → reduces endogenous oxalate synthesis	IL-6 mediates oxidative stress (e.g., 8-OHdG↑) → promotes the inflammatory microenvironment → crystal adhesion
Core mechanics	Metabolic regulation: Reduces oxalic acid production	Inflammatory damage: oxidative stress exacerbates tubular epithelial damage
Biomarkers	DAO expression ↓, serum/urine oxalate level ↓	8-OHdG↑, MDA↑, SOD activity↓
Functional positioning	Inhibition of upstream oxalic acid synthesis	Downstream crystal deposition facilitation

In the formation of calcium oxalate kidney stones, IL-6 shows a dual regulation of “early protection - late injury”: In the early stage, it induces methylation of the DAO gene through the JAK/STAT3 pathway and inhibits oxalate synthesis; In the late stage, the ROS/NF-κB pathway is activated under the drive of chronic inflammation, and the increased oxidative stress promotes crystal deposition.

### 2.2 Emerging roles of non-classical interleukins in calcium oxalate nephrolithiasis

Beyond IL-1β and IL-6, emerging evidence highlights the involvement of other interleukin family members in CaOx renal stone pathogenesis. Clinical data analyses reveal significantly elevated serum IL-11 levels in idiopathic Calcium Oxalate Nephrolithiasis patients. Mechanistic investigations propose its potential engagement in lithogenesis, possibly through orchestrating chemokine cascade interactions and activating Toll-like receptor (TLR)/NOD-like receptor (NLR) signaling pathways (25). Furthermore, investigations along the gut microbiota-kidney axis demonstrate that intestinal dysbiosis increases nephrolithiasis susceptibility via IL-18 upregulation (26), revealing novel inflammatory cytokine-mediated crosstalk in gut-renal pathophysiology (**Figure 1**). Nevertheless, research on non-classical pro-inflammatory interleukins remains nascent, with critical knowledge gaps persisting in three domains: (1) signaling specificity regarding detailed regulatory mechanisms of their activated pathways; (2) cytokine synergy involving combinatorial effects with other inflammatory mediators; and (3) systems-level integration of cross-talk between interleukin networks and metabolic reprogramming. Future studies should employ multi-omics strategies, particularly integrated metabolomic-proteomic analyses, to systematically map interleukin-mediated inflammatory networks and their spatial-temporal interactions in CaOx stone microenvironments. Such approaches will advance our mechanistic understanding beyond isolated cytokine observations toward comprehensive pathway deciphering.

### 2.3 Advances and challenges of anti-inflammatory factors in calcium oxalate kidney stone formation

The inflammatory microenvironment represents a dynamic equilibrium between pro-inflammatory and anti-inflammatory factors. In the field of anti-inflammatory regulation, IL-4 and IL-13 have been shown to stimulate macrophage polarization toward the M2 phenotype, enhancing their capacity to phagocytose calcium oxalate crystals and reducing oxidative stress damage in human renal tubular epithelial cells (HK-2) (27). Notably, emerging research highlights the critical protective role of IL-10. Experimental evidence demonstrates that oxalate-rich microenvironments significantly suppress IL-10 expression, while exogenous IL-10 supplementation effectively reverses oxalate-induced monocyte/macrophage dysfunction. Furthermore, a recent study demonstrated that IL-10-secreting constitutive RAW264.7 macrophages attenuate calcium oxalate crystal adhesion and deposition in both murine models of Calcium Oxalate Nephrolithiasis and *in vitro* models of COM-induced renal injury, mechanistically through downregulation of osteopontin (OPN) expression. This suggests that targeting the IL-10 signaling pathway may represent a novel therapeutic strategy for preventing and treating Calcium Oxalate Nephrolithiasis (28, 29).These findings elucidate the mechanisms underlying anti-inflammatory factor-mediated macrophage-epithelial cell interactions, providing a theoretical foundation for developing multi-target synergistic anti-lithiasis therapies (**Figure 1**).

Although these studies preliminarily reveal the regulatory roles of anti-inflammatory factors in calcium oxalate stone formation, systematic investigations into their involvement remain scarce compared to the well-characterized pro-inflammatory cytokine network. Other cytokines with potential anti-inflammatory properties, including IL-37 and IL-1RA, play key negative regulatory roles in the inflammatory response during gout crystal formation. It is worth noting that IL-37 promotes its own nuclear translocation by forming a complex with phosphorylated Smad3, thereby inhibiting the NLRP3–caspase-1–IL-1β signaling axis and mediating its anti-inflammatory effects. IL-1Ra mainly negatively regulates gout-associated inflammation by competitively inhibiting the IL-1β signaling pathway (30, 31). Currently, the roles of IL-37 and IL-1RA in the calcium oxalate crystal microenvironment remain unclear, and related studies are relatively limited. Inspired by their known functions in the gout inflammatory pathway, further investigation into the specific mechanisms of these two cytokines in calcium oxalate crystal-induced inflammation may provide new research directions and theoretical breakthroughs in this field.

## 3 Research status of chemokines in calcium oxalate nephrolithiasis

### 3.1 Overview of chemokines

Chemokines are small secreted proteins that interact with G protein-coupled receptors (GPCRs) on cell surfaces to stimulate cellular migration, particularly the directional movement of leukocytes. Based on the arrangement of cysteine (Cys) residues in their structures, chemokines are classified into four subfamilies: CC, CXC, CX3C, and XC. Beyond guiding cell migration, chemokines participate in diverse biological processes, including cell proliferation, survival, differentiation, and cytokine production. They play essential roles in immune system development, homeostasis maintenance, and immune response regulation (32, 33).

### 3.2 Structural and receptor-mediated mechanisms of MCP-1/CCL2 in inflammatory diseases

Monocyte chemoattractant protein-1 (MCP-1/CCL2), a key member of the CC chemokine subfamily, is a potent chemoattractant for monocytes. The MCP-1 gene is located on chromosome 17q11.2–q12 and encodes a 76-amino acid protein with a molecular weight of 13 kDa. Structurally, MCP-1 comprises a flexible N-terminal region, a long loop, and an α-helix, with the N-terminal domain being critical for receptor binding and activation. MCP-1 is expressed in various cell types, including endothelial cells, epithelial cells, and tumor cells. Its specific receptor, CCR2—a GPCR predominantly expressed on monocytes and T lymphocytes—activates downstream signaling pathways upon MCP-1 binding, recruiting immune cells such as monocytes/macrophages to inflammatory sites. This mechanism may contribute to the pathogenesis of diseases such as asthma, atherosclerosis, and breast cancer, potentially through amplified inflammatory signaling mediated by monocyte recruitment and cytokine release (34, 35). The MCP-1/CCR2 axis is also pivotal in renal pathologies. In nephritis, renal injury, kidney failure, and renal carcinoma, MCP-1 exacerbates inflammatory responses by promoting monocyte and macrophage infiltration, with its expression levels correlating strongly with the severity of renal damage (36). In diabetic nephropathy, MCP-1 acts as a critical inflammatory mediator, regulating inflammatory cascades and accelerating disease progression via oxidative stress activation (37–40).

### 3.3 Molecular mechanisms of MCP-1 in driving nephrolithiasis formation

#### 3.3.1 Expression characteristics of MCP-1 in nephrolithiasis

**Figure 2:**
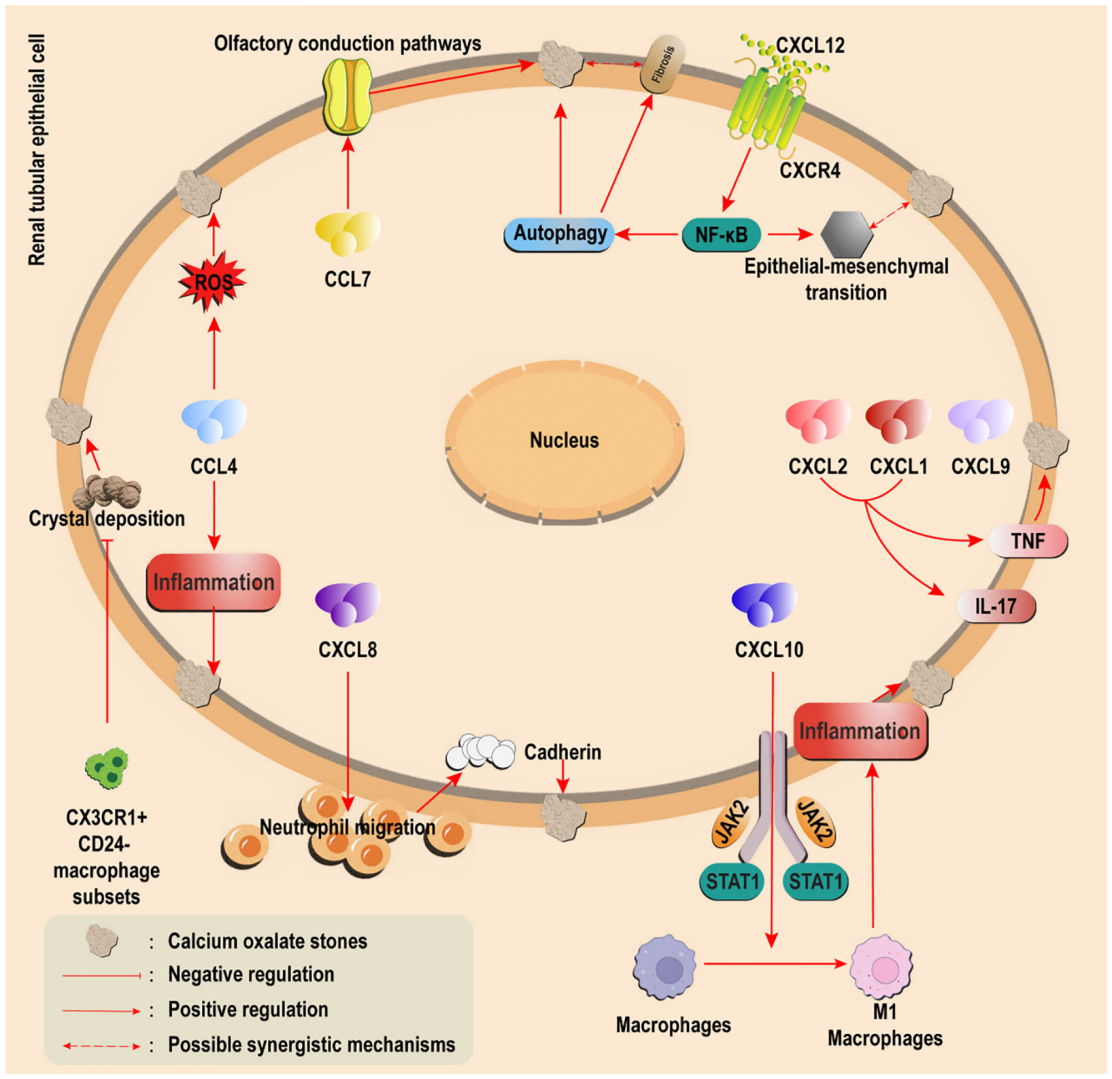
Regulatory effects of other chemokines on calcium oxalate stones. Overexpression of CCL5/7 (CCL7 or through the olfactory pathway) Pathogenic CXCL1/2/8/9/10 was upregulated (CXCL8 promotes inflammation and retention, and CXCL10 drives M1 polarization); Protective CXCL14 induces M2 polarization to promote crystal clearance; The CXCL12/CXCR4 axis drives autophagy/EMT via NF-κB and connects stones with fibrosis. CX3CR1+CD24− macrophages inhibit crystal adhesion.

**Figure 3:**
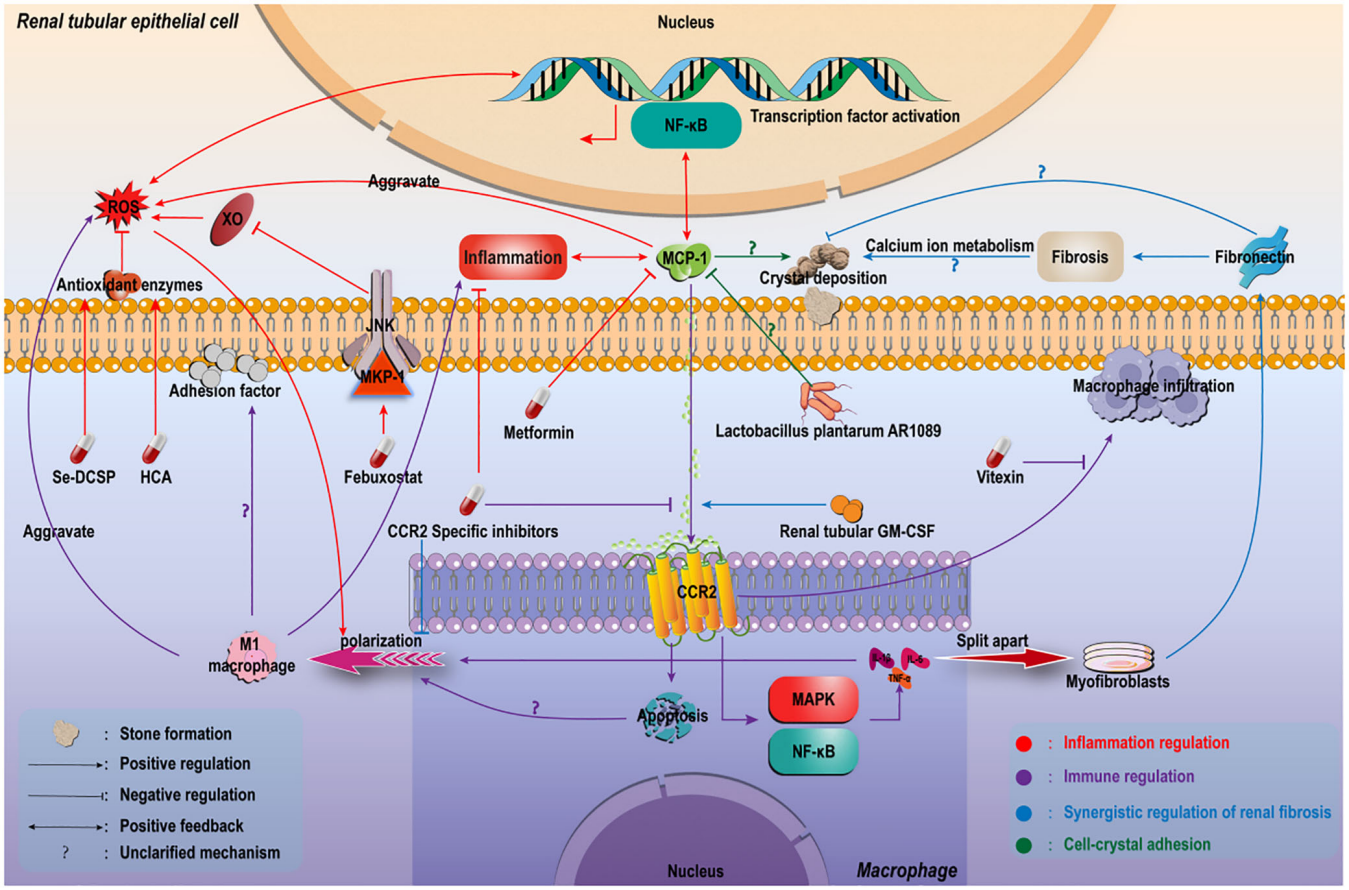
Regulatory pathway of MCP-1 in Calcium Oxalate Nephrolithiasis formation. CaOx crystals induce high expression of MCP-1. MCP-1/CCR2 axis: 1) Drives the recruitment and activation of M1 macrophages, releases inflammatory factors/ROS, and damages renal tubules; 2) Enhance crystal adhesion; 3) Promote fibrosis. The fibrotic microenvironment promotes stones in reverse. This axis is the core hub and potential target.

Multiple clinical studies have demonstrated significantly elevated levels of MCP-1 and CCR2 in the urine and renal papillae of kidney stone patients (41, 42). Additionally, stone composition analysis revealed prominent MCP-1 expression in renal tissues of CaOx stone formers, suggesting its potential involvement in crystal adhesion and inflammatory microenvironment formation (43). In ethylene glycol-induced rat models of Calcium Oxalate Nephrolithiasis, MCP-1, CXCL1, and CXCL2 are released, activating TNF and IL-17 signaling pathways, which may play pivotal roles in stone pathogenesis (44, 45) (**Figure 2**). In murine CaOx stone models, intraperitoneal glyoxylate administration upregulated MCP-1 expression, accompanied by macrophage infiltration and tubular injury. Notably, CCR2 inhibition significantly reduced crystal deposition and ameliorated renal function (46) (**Figure 3**).

#### 3.3.2 Synergistic effects of MCP-1 in directly mediating inflammation and oxidative stress

Accumulating evidence indicates that MCP-1 promotes the pathological progression of nephrolithiasis by orchestrating inflammatory and oxidative stress mechanisms. Experimental studies demonstrate that selenium-modified corn silk polysaccharides significantly alleviate oxidative damage in renal tubular epithelial cells by suppressing MCP-1 expression induced by CaOx crystals (47). Similarly, febuxostat reduces ROS generation through modulation of the MCP-1 signaling pathway, thereby inhibiting CaOx crystal-induced oxidative stress (48). Additionally, compounds such as hydroxycitrate and metformin have been shown to downregulate MCP-1 expression, exerting anti-inflammatory and antioxidant effects that reduce renal crystal deposition (49, 50). These findings collectively suggest that MCP-1 facilitates CaOx crystallization by directly activating synergistic interactions between inflammatory responses and oxidative stress, thereby establishing a favorable microenvironment for stone formation (**Figure 3**).

#### 3.3.3 Immunoregulatory mechanisms of macrophage polarization

M1-polarized macrophages are regarded as contributing factors to nephrolithiasis due to their role in exacerbating inflammatory injury and oxidative stress (14). Recent studies have further revealed that MCP-1 modulates stone progression by regulating immune cell phenotypes. In CaOx crystal stimulation models, MCP-1 binding to its receptor CCR2 significantly promotes macrophage polarization toward the M1 phenotype, a process closely associated with renal oxidative stress and apoptosis levels. Specific inhibition of CCR2 effectively reverses M1 polarization and alleviates crystal-induced inflammatory damage (46). Notably, the flavonoid compound Vitexin demonstrates marked renoprotective effects by suppressing MCP-1-mediated macrophage infiltration (51). These findings confirm that MCP-1 not only mediates immune cell chemotaxis but also amplifies inflammatory signaling by reshaping macrophage functional phenotypes, thereby fostering a vicious cycle that promotes stone development (**Figure 3**).

#### 3.3.4 Molecular regulation of crystal adhesion

The adhesion of crystals to renal tubular epithelia represents a critical step in stone formation. Recent studies demonstrate a positive correlation between MCP-1 expression levels and the extent of crystal deposition. Lactiplantibacillus plantarum AR1089 significantly inhibits renal crystallization by downregulating MCP-1 and related adhesion molecule expression (52). Mechanistic investigations confirm that MCP-1 synergizes with molecules such as osteopontin (OPN) to enhance CaOx crystal adhesion to renal tubular cells (53). However, the potential cooperative mechanism of these two factors has not been specifically clarified. The current evidence is limited to their parallel overexpression during the formation of calcium oxalate stones and their promotion of formation. Furthermore, in vascular endothelial cell models, suppression of MCP-1 effectively reduces intercellular adhesion molecule activity, suggesting that MCP-1 may similarly modulate crystal adhesion properties in tubular epithelia (54). Emerging studies further indicate that MCP-1 may indirectly influence crystal deposition through immune cell-mediated mechanisms. Activation of M1-polarized macrophages promotes adhesion-related gene expression, creating a favorable microenvironment for crystal retention (55). These findings highlight the need to investigate how MCP-1-regulated M1 macrophage polarization modulates crystal-cell interactions, which may provide novel perspectives for understanding nephrolithiasis pathogenesis (**Figure 3**).

#### 3.3.5 MCP-1 as a potential regulatory hub linking renal fibrosis and nephrolithiasis

Beyond directly mediating inflammatory responses, chemokines such as MCP-1 may indirectly influence stone formation by regulating fibrotic processes (**Figure 3**). The interplay between renal fibrosis and nephrolithiasis has emerged as a research focus, though their intrinsic relationship remains incompletely elucidated. This interaction involves multifactorial regulation. Studies indicate that fibronectin, a hallmark of renal fibrosis, exhibits expression levels positively correlated with fibrotic progression (56, 57). Meanwhile, the MCP-1/CCR2 axis promotes renal interstitial fibrosis by recruiting inflammatory cells and inducing macrophage-to-myofibroblast transdifferentiation (58, 59). Despite their shared involvement in fibrosis, the direct regulatory relationship between MCP-1 and fibronectin requires validation through co-expression analyses or genetic editing models. Notably, inhibition of the MCP-1 pathway concurrently ameliorates both fibrosis and stone formation (60, 61),underscoring its potential as a dual therapeutic target.

Intriguingly, fibronectin demonstrates dual roles: it may facilitate stone formation by promoting crystal aggregation and extracellular matrix invasion, while simultaneously inhibiting CaOx crystal nucleation and adhesion on renal tubular surfaces (62) (**Figure 3**). Current debates persist regarding the causality between fibrosis and stone formation. Some clinical studies suggest that fibrosis in stone patients may be driven by alternative pathways, such as STAT6 and ferroptosis signaling (63, 64). In ischemia-reperfusion injury models, tubular GM-CSF upregulates the MCP-1/CCR2 axis to drive advanced fibrosis (65), implying MCP-1 may act as a downstream effector of other mediators. Collectively, existing evidence indicates pathophysiological interactions between these processes: renal fibrosis promotes crystal formation by disrupting calcium ion metabolism, while fibrotic microenvironments strengthen crystal-cell interactions, thereby providing favorable conditions for stone development.

Future research should utilize conditional MCP-1 knockout mouse models to clarify the temporal sequence between fibrosis and stone formation. Furthermore, clinical analyses assessing the dynamic changes in MCP-1 levels and their correlation with fibrosis progression in stone patients are essential to validate the potential bridging role of the MCP-1/CCR2 signaling axis. The bidirectional regulatory properties of MCP-1 provide new research perspectives for unraveling the intricate network linking renal fibrosis and nephrolithiasis, while also opening up potential avenues for exploring pathological mechanisms and therapeutic strategies.

### 3.4 Multidimensional regulation by the chemokine family in the pathological progression of nephrolithiasis

Beyond MCP-1, other chemokines exhibit complex regulatory networks during CaOx stone formation (**Figure 2**). Although CCL4 has been implicated in mediating inflammatory injury and oxidative stress (66), its specific role in nephrolithiasis remains to be fully elucidated. Notably, CCL5 and CCL7 show marked overexpression in CaOx stone patients (41, 67), with CCL7 potentially regulating crystal formation through olfactory signaling pathways (68).

Within the CXC chemokine subfamily, CXCL1, CXCL2, CXCL8 (IL-8), CXCL9, and CXCL10 are significantly upregulated in CaOx stone models and play pivotal roles in pathogenesis (44, 69). Among these, CXCL8 is the most well-characterized: it enhances neutrophil migration to amplify inflammation and upregulates adhesion molecules such as cadherins on renal tubular epithelial cells, directly promoting crystal retention and deposition. Additionally, CXCL8-mediated cadherin modulation may remodel the stone-forming microenvironment (70). Other CXC members, including CXCL10, drive macrophage M1 polarization via JAK2/STAT1 pathway activation, exacerbating renal inflammation (71). In contrast, CXCL14—elevated in high-fat diet murine models—induces macrophage M2 polarization, enhancing phagocytic clearance of crystals on bladder epithelial surfaces and thereby inhibiting pathological stone progression (72), this finding highlights CXCL14 as a promising target for Calcium Oxalate Nephrolithiasis research.

The CXCL12/CXCR4 axis, where CXCL12 is the sole ligand for CXCR4, has recently emerged as a critical hub linking stone formation to fibrosis. By activating the NF-κB pathway, this axis drives autophagy and epithelial-mesenchymal transition (EMT), directly contributing to stone-related renal injury while establishing a molecular bridge between crystal deposition and fibrotic progression (73).Further investigation of this mechanism may clarify chemokines’ dual roles in secondary stone injury and reveal CXCL12’s underappreciated contributions to Calcium Oxalate Nephrolithiasis.

Finally, the CX3CR1+CD24− macrophage subset demonstrates unique protective functions by suppressing crystal adhesion mechanisms, offering novel insights into preventive strategies for CaOx stone formation (74).

## 4 The IL-6/MCP-1/miR-124-3p negative feedback regulatory network

**Figure 4:**
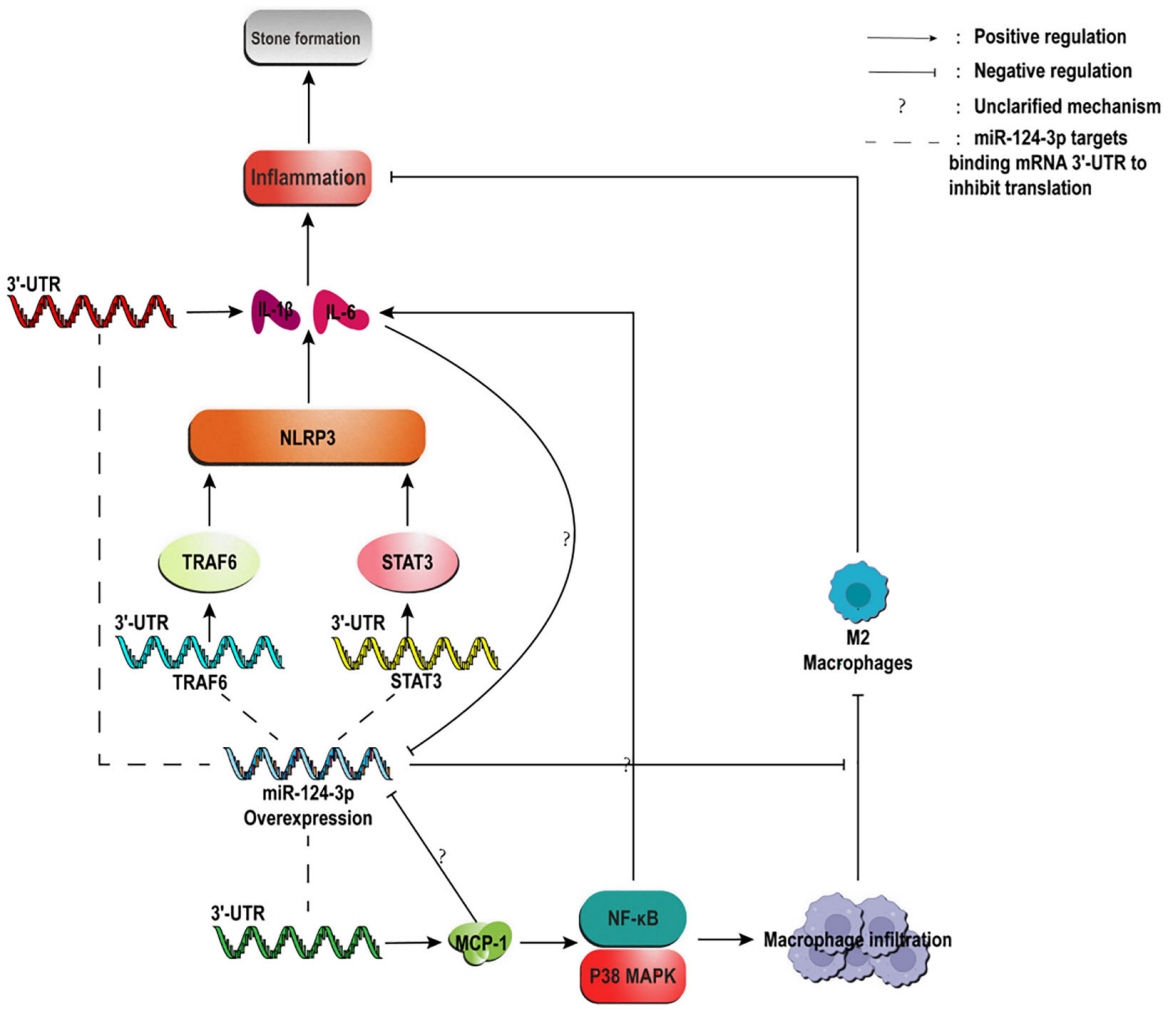
Possible mechanisms by which IL-1βIL-6/MCP-1 regulates calcium oxalate stone formation by regulating miR-124-3p. Under pathological conditions, overexpressed IL-6 and MCP-1 inhibit the anti-inflammatory molecule miR-124-3p. The reduction of miR-124-3p leads to its inability to effectively inhibit the key targets (TRAF6/STAT3/MCP-1) and downstream pathways (NF-κB/MAPK/NLRP3), and simultaneously weakens the ability to promote M2 polarization in macrophages. Together, it aggravates the inflammatory response, deteriorates the microenvironment, and promotes the deposition of CaOx crystals and the formation of stones. Form a self-perpetuating vicious cycle, highlighting the core hub position and therapeutic potential of miR-124-3p.

In recent years, the regulatory roles of non-coding RNAs in inflammatory microenvironments have garnered increasing attention. Emerging studies reveal that miR-124-3p directly binds to the 3’-untranslated regions (3’-UTRs) of TRAF6 and STAT3, suppressing NLRP3 inflammasome activation and downstream inflammatory signaling, thereby significantly inhibiting the protein expression of key pro-inflammatory cytokines such as IL-6 and IL-1β (75, 76). Overexpression of miR-124-3p also attenuates NF-κB and p38 MAPK signaling pathway activity, reducing pro-inflammatory factor expression and alleviating inflammatory responses (77, 78). Notably, miR-124-3p has been shown to directly target and regulate interleukin family members (79). In Calcium Oxalate Nephrolithiasis, miR-124-3p expression is downregulated alongside MCP-1 upregulation. Mechanistically, miR-124-3p inhibits MCP-1 expression by targeting its 3’-UTR (80, 81). Furthermore, miR-124-3p is closely linked to macrophage polarization, suppressing macrophage migration in inflammatory environments and promoting their transition to the M2 phenotype (82, 83). These findings suggest that miR-124-3p may serve as a molecular hub connecting inflammatory responses to stone formation through multi-target regulation of inflammatory networks, offering new avenues for miRNA-based targeted therapies (**Figure 4**).

Building on the multi-target nature of miR-124-3p, we propose the “IL-6/MCP-1/miR-124-3p negative feedback loop” hypothesis: In CaOx crystal-rich environments, overexpressed IL-6 and MCP-1 suppress miR-124-3p expression via negative feedback. This dual mechanism (1) diminishes miR-124-3p’s inhibitory effects on STAT3/NF-κB/MAPK pathways, amplifying pro-inflammatory cytokine release and exacerbating inflammation; and (2) reverses anti-inflammatory macrophage polarization, thereby promoting CaOx stone formation. Future studies should employ miR-124-3p-specific knockout models to assess its impact on stone susceptibility and cytokine profiles. Additionally, this regulatory loop highlights the potential for synergistic therapies combining miR-124-3p mimics or exosome-based delivery systems with existing anti-inflammatory agents. In-depth investigation of this closed-loop regulatory network is expected to advance our understanding of the molecular mechanisms underlying Calcium Oxalate Nephrolithiasis and identify novel research directions.

## 5 Conclusions and perspectives

The critical role of inflammatory microenvironments in driving Calcium Oxalate Nephrolithiasis has been firmly established. This review comprehensively outlines the molecular mechanisms by which interleukin and chemokine families promote stone formation through inflammation, oxidative stress, crystal adhesion, and macrophage polarization. These insights not only refine our understanding of nephrolithiasis pathogenesis but also highlight potential therapeutic targets for clinical intervention. Emerging evidence further underscores the interplay between renal fibrosis and stone formation, with MCP-1 serving as a pivotal mediator. Building on this, we propose the “fibrosis-stone vicious cycle” hypothesis, wherein CaOx crystals activate the MCP-1/CCR2 axis to recruit pro-inflammatory M1 macrophages, which release fibrogenic factors like TGF-β1, accelerating renal fibrosis. Conversely, fibrotic microenvironments disrupt calcium metabolism and extracellular matrix dynamics, fostering crystal retention. Validating this hypothesis necessitates combinatorial therapies (e.g., CCR2 antagonists with anti-fibrotic agents) and longitudinal clinical studies tracking urinary MCP-1 levels alongside fibrosis biomarkers (e.g., α-SMA, collagen III). While this model offers a theoretical foundation for dual therapeutic targeting, its feasibility requires validation in advanced experimental systems such as organoids or humanized mice.

Therapeutic strategies targeting inflammatory pathways have gained momentum. Monotherapies against pro-inflammatory cytokines or supplementation of anti-inflammatory factors have demonstrated efficacy in modulating stone microenvironments, with preclinical studies showing promising anti-lithogenic effects. As previously highlighted, IL-10-engineered macrophage transplantation is an emerging therapeutic strategy that can effectively promote the clearance of calcium oxalate kidney stones and alleviate related renal tubular damage (29). In addition, chloroquine-containing nano-selenium and Burttet Hill (CA) suppress NLRP3 expression by inhibiting NF-κB pathway phosphorylation, thereby reducing secretion of IL-1β and IL-6 as well as OPN expression, which minimizes crystal deposition and kidney damage (84, 85). Roxadustat, a proline hydroxylase domain inhibitor,significantly lowers MCP-1 and OPN levels both *in vivo* and *in vitro*, inhibiting calcium oxalate stone formation (86). Furthermore, CCR2 antagonists alleviate the damage of HK-2 cells *in vivo* and *in vitro* by inhibiting CaOx-induced M1 polarization of macrophages and reducing the expression of inflammatory markers IL-1β, IL-6 and MCP-1, thereby reducing renal oxidative stress, inflammation and apoptosis (46). However, clinical evidence for these novel strategies remains limited, and human trials are still scarce. Future research should focus on prospective randomized controlled trials in kidney stone patients, evaluating efficacy and safety through stone recurrence rates and urinary inflammatory biomarkers. Such efforts will facilitate the translation of these approaches from bench to bedside. Future innovations may include involve dual-target systems (e.g., CCR2 antagonists combined with miR-124-3p agonists) to simultaneously suppress inflammation and enhance crystal clearance. Integrating gut microbiota modulation with cytokine-targeted therapies could further reduce recurrence risks, while biomarker discovery may enable early risk stratification and treatment monitoring.

Despite significant advances in understanding the roles of interleukins and chemokines in nephrolithiasis, critical challenges remain unresolved. The specific contributions of anti-inflammatory interleukins (e.g., IL-37, IL-1RA) and non-canonical pro-inflammatory interleukins (e.g., IL-11, IL-18) to stone formation are incompletely elucidated, particularly their interactions with the gut microbiota-kidney axis, which demand further exploration. Additionally, the synergistic effects of chemokines such as CXCL12/CXCR4 with inflammatory, oxidative stress, and fibrotic pathways remain ambiguous. Future studies should integrate multi-omics technologies to delineate their regulatory networks, supplemented by longitudinal clinical cohort analyses to strengthen causal evidence linking chemokines to fibrosis. Translational hurdles persist, as the safety and efficacy of existing targeted therapies require systematic validation. Further research should also focus on refining molecular mechanisms, including the detailed roles of anti-inflammatory factors like IL-10 in suppressing crystal deposition via macrophage phenotype modulation, as well as elucidating structure-activity relationships of chemokines at crystal-cell interfaces. Notably, while the development of multi-pathway combination therapies and elucidation of the regulatory roles of the miR-124-3p-centered inflammatory network in Calcium Oxalate Nephrolithiasis hold significant potential for advancing prevention and treatment, they concurrently present substantial scientific and translational challenges.

In summary, interleukins and chemokines orchestrate Calcium Oxalate Nephrolithiasis via multifaceted inflammatory and fibrotic networks. The proposed IL-6 dynamic regulation, fibrosis-stone vicious cycle, and IL-6/MCP-1/miR-124-3p feedback loop hypotheses provide novel frameworks to address current research bottlenecks. Future studies should employ interdisciplinary approaches to validate these models, explore the cooperative mechanisms between interleukins and chemokine families in depth, and advance precision therapeutic strategies targeting inflammatory microenvironments, thereby offering new directions for the prevention and management of nephrolithiasis.
